# How Parents Perceive the Potential Risk of a Child-Dog Interaction

**DOI:** 10.3390/ijerph19010564

**Published:** 2022-01-05

**Authors:** Jan Náhlík, Petra Eretová, Helena Chaloupková, Hana Vostrá-Vydrová, Naděžda Fiala Šebková, Jan Trávníček

**Affiliations:** 1 Department of Animal Husbandry Sciences, Faculty of Agriculture, University of South Bohemia in Ceske Budejovice, Studentská 1668, 370 05 Ceske Budejovice, Czech Republic; mvdrnahlik@seznam.cz (J.N.); travnic@zf.jcu.cz (J.T.); 2 Department of Ethology and Companion Animal Science, Faculty of Agrobiology Food and Natural Resources, Czech University of Life Sciences Prague, Kamýcká 129, 165 00 Prague, Czech Republic; chaloupkovah@af.czu.cz (H.C.); vostrah@af.czu.cz (H.V.-V.); sebkova@af.czu.cz (N.F.Š.)

**Keywords:** dog, dog-child interaction, breed, danger assessment, online survey, dog attack

## Abstract

Dog attacks on children are a widespread problem, which can occur when parents fail to realise a potentially dangerous interaction between a dog and a child. The aim of the study was to evaluate the ability of parents to identify dangerous situations from several everyday child–dog interactions and to determine whether the participants connected these situations to a particular breed of dog. Five sets of photographs depicting potentially dangerous interactions from everyday situations between children and three dogs (one of each breed) were presented via an online survey to parents of children no more than 6 years old. Data from 207 respondents were analysed using proc GLIMMIX in SAS program, version 9.3. The probability of risk assessment varied according to dog breed (*p* < 0.001) as well as to the depicted situation (*p* < 0.001). Results indicated that Labrador Retriever was considered the least likely of the three dogs to be involved in a dangerous dog-child interaction (with 49% predicting a dangerous interaction), followed by Parson Russell Terrier (63.2%) and American Pit Bull Terrier (65%). Participants considered one particular dog-child interaction named ‘touching a bowl’ a dangerous interaction at a high rate (77.9%) when compared with the other presented situations, which were assessed as dangerous at rates of 48.4% to 56.5%. The breed of dog seems to be an influential factor when assessing a potentially dangerous outcome from a dog-child interaction. Contrary to our hypothesis, interactions involving the small dog (Russell Terrier) were rated more critically, similarly to those of the Pit Bull Terrier. These results suggest that even popular family dog breeds, such as Labrador Retrievers, should be treated with more caution.

## 1. Introduction

The close relationship of man to dog often leads to tendencies towards anthropomorphism [1], tolerance towards inappropriate behaviour [2], and underestimating high-risk yet everyday situations [3]. All of the above can lead to a number of problems, ranging from subtle displays of aggressive behaviour by dogs towards humans [4] to very serious attacks against children [5]. Children between 5 and 9 years of age comprise the bulk of dog bite victims worldwide: Based on hospital admissions for surgical treatment, this trend has been observed in Canada [5], the United States [6,7], Austria [8], Italy [9] Turkey [10] and the Czech Republic [11]. Children are usually bitten on the head, particularly on the face, hands, neck, or throat [5,9,12,13,14], and these injuries may lead to a lifetime of physical impairment for the victims and considerable financial strain for parents in the form of medical expenses [15]. The most serious attacks result in the death of the victim and often the eventual abandonment or euthanasia of the animal [16,17].

Generally, aversive and conflict-escalating signals by dogs can be easily interpreted by adults [18,19,20]; however, signals intended to avoid or defuse conflict are much harder to identify for both adults and children [21,22,23]. Children younger than 6 years of age are unable to recognise audio–visual signals by dogs in a reliable manner [22]. The progression of changes in a dog’s behaviour, from conflict-defusing (i.e., yawning, turning head away, nose licking) and conflict-avoiding (walking away, tucking tail) signals to conflict-escalating (growling, snapping) indicators, coined by Shepherd [24] as the ‘ladder of aggression’, can occur quickly. Overlooking or misinterpreting these signals could lead to a biting incident. Adult guardian supervision should be considered an important factor to ensure safe interaction between the dog and the child [25], although guardians often underestimate the dangers of child–dog interactions and many leave their children unattended with the family dog for short periods of time [3]. This single study also reported that survey participants tend to ignore expert advice and continue to allow these potentially dangerous interactions to occur because they believe their dogs are not aggressive [3]. Several studies report that educational courses have been established internationally in order to teach children and parents alike about the potential dangers of interactions with dogs [26,27]; however, the long-term benefits of a number of such programs have been shown to be ineffective in preventing child injury and would not stop parents from allowing their children to engage in risky behaviour when interacting with unfamiliar dogs [28,29]. However, one program that examined the natural ability of children and parents to identify conflict signalling and provided both groups with useful knowledge showed greater promise in the long-term prevention of dog-related child injuries [30]. Even if children receive instruction on how to act in the presence of dogs, many attacks occur when the child engages in certain behaviours. Examples of this might include a child attempting to play with the animal, trying to take its toy or touching its bowl when the dog is feeding, entering a space occupied by the dog, attempting to hug the animal around its neck, or suddenly awakening or startling the animal [3,11,31,32]. Arhant et al. [33] indicate that even generally positive activities such as playing or feeding treats to the dog may not be completely safe.

A recent US-based study analysed how several small animal veterinarians perceived aggression in common breeds [34]; results indicated that regarding bite severity (bites that require medical treatment), Labrador Retriever was perceived as posing the lowest risk, followed by Jack Russell Terrier and Pit Bull (moderate risk) and finally the German Shepherd, Chow Chow, and Chihuahua (high risk). On the other hand, the participants of this study agreed that all breeds are equally likely to bite a person. The question arises as to whether parents of young children assess dog-child interactions based on the dog breed or size.

There is a substantial worldwide amount of evidence (e.g., [31,34,35,36,37,38,39]) indicating that the following breeds are most commonly involved in biting incidents targeting humans: Dachshunds, German Shepherds, Rottweilers, and Bull-type breeds such as Pit Bull Terriers. Small dogs, such as the Jack Russell Terrier group (consisting of the Jack Russell Terrier [40] and Parson Russell Terrier [41] as recognised by CMKU, the Czech branch of Fédération cynologique internationale (FCI)), are excitable and more prone to respond aggressively towards humans (including household members [35]) or other dogs than are some larger breeds [32,35,42,43]. Pit Bull Terriers are reported to be more likely to bite strangers unprovoked [44]. Their bites are more likely to require medical attention than those of other dogs [44,45]. Pit Bull attacks gain substantial media attention, which often results in an unfavourable reputation for the breed [45,46]. Moreover, several studies [5,8,31,43] have reported that even Labradors or Golden Retrievers have been recorded in biting incident statistics, despite being highly regarded as family pets. At the same time, bites from Labradors or Golden Retrievers rarely result in serious injury, unlike the Rottweiler or German Shepherd, which are more frequently involved in serious or lethal biting incidents [31,35,38,39], albeit less frequently than Pit Bull Terriers [35,44]. However, these studies were carried out in areas with a high proportion of Labrador or Golden Retrievers, and therefore, their overrepresentation in biting incidents could be misleading. Additionally, cross-breeds or unregistered dogs are responsible for a large percentage of human-directed biting incidents [5,8,31,36,38]. Records show that Jack Russell Terriers and Pit Bull Terriers frequently display aggressive behaviour towards strangers and other dogs [35]. Studies from many countries indicate that the majority of child victims of biting incidents are bitten by dogs of their own household or dogs familiar to their family members [5,8,31,36,38,47].

The risk factors leading to dog attacks on children as described above are documented in the Czech Republic [11,47] as well as worldwide [3,8,28,31,48,49]; however, it is unclear whether parents are aware of the data and to what extent they refer to them when their children come into contact with dogs. Additionally, dog ownership may be an advantage when it comes to adults recognising dog cues [21], but whether dog owners are more adept at recognising dangerous situations remains unknown.

The objective of this study was to determine the following: (1) how parents of children aged 6 years or younger recognise potentially dangerous interactions between a child and a dog; (2) whether participants attach importance to individual dog breeds when determining the degree of danger in a presented situation; (3) whether dog ownership increases the probability of identifying a dangerous interaction. We hypothesised that respondents would not perceive all situations as potentially dangerous and that they would view situations involving a small dog (Parson Russell Terrier) or a ‘family recommended’ dog (Labrador Retriever) as less dangerous than those involving a Pit Bull Terrier.

## 2. Materials and Methods

### 2.1. Ethical Approval

The project was approved by the Guide for the Care and Use of Animals of the Czech University of Life Sciences Prague. The study and its methodological procedure adhered with the requirements of the European Union and Czech legislation (Act No. 246/1992 Coll. on animal protection as amended by Act No. 162/1993 Coll.). The dog owners gave their explicit written consent for their dogs to participate in the study. They were properly informed of the experimental design, and they also affirmed that their dogs had no history of attacks towards children. In order to minimise risk, only healthy, docile dogs that were familiar with the child participant were selected. The parents provided written consent for their child to participate in the study following a detailed explanation of the study design. At least one parent was present while the photographs were being taken.

### 2.2. Materials and Dogs

Veterinarians from the database of the Veterinary Clinic Bohemia (Pisek, Czech Republic) selected only healthy, vaccinated, dewormed, and obedient dogs for involvement in this study. The survey consisted of photographs of a 6-year-old girl in every day, though potentially dangerous, situations with 3 different dogs. Participants were asked to rate each photograph (the child interacting with all three dogs separately) for the level of potential danger. The dogs were selected from a pool that comprised 37 Labrador Retrievers, 12 Parson Russell Terriers, and 3 American Pit Bull Terriers. Dog owners were contacted by phone and informed about the experimental design, and they also affirmed that their dogs had no history of attacks towards children. Prior to the photo shoot, veterinarian Dr. Jan Nahlik examined the animals and tested their basic obedience at the Veterinary Clinic Bohemia, in order to select 3 ideal dogs that would participate in the study.

The 6-year-old girl in the photos regularly participates in dog and puppy training activities. She was instructed on how to react if the dogs behaved aggressively towards her during the photo shoot. The parents provided written consent for their child to participate in the study following a detailed explanation of the study design, and at least one parent was present during the entire procedure. The sets of photographs were taken by a dog behaviourist (veterinarian) who also evaluated the animals and individual situations.

The five situations captured in the photos are everyday interactions that routinely occur between dogs and children but are also known to trigger adverse reactions in dogs. The task was to mark which photographs for each situation, and each dog presented a potentially dangerous interaction. The following dogs participated in the experiment: (1) an 8-year-old castrated male Labrador Retriever (hereinafter referred to as the ‘Labrador’) crossbreed as a representative of a ‘family recommended’ dog; (2) a 9-year-old neutered female American Pit Bull Terrier (hereinafter referred to as the ‘Pit Bull’) as a representative of a ‘problematic dog’; (3) a 12-year-old neutered female Parson Russell Terrier (hereinafter referred to as the ‘Russell’) as a representative of a ‘small dog’. According to the Czech studbook (CMKU, [50]), the Labrador Retriever is one of the most popular breeds overall. Among small-sized terriers, the Parson and Jack Russell are the two most favoured breeds. Although the American Pit Bull Terrier is not recognised by the FCI, it is bred in, at least, one non-FCI Czech breeding club [51]. In the Czech Republic, public opinion regarding the American Pit Bull varies between overwhelmingly positive (regarded by some as an ideal pet) [51,52] and scathingly negative (viewed by others as aggressive and uncontrollable; with a reputation for biting humans) [53,54,55]. Ownership and breeding of the Pit Bull Terrier are not limited by any legislation in the Czech Republic.

### 2.3. Data Collection

Data collection occurred between September 2019 and October 2019. An online internet questionnaire (in Czech) was created using the website Survio (https://www.survio.com/cs/) (accessed on 1 September 2019). Respondents were contacted and invited to complete the questionnaire either in person, through the social media platform Facebook (via the Veterinary Clinic Bohemia profile page), or by email.

A total of 492 individuals visited the website; of those, 284 respondents merely viewed the questionnaire, while 207 respondents completed the online form.

### 2.4. Questionnaire

The questionnaire was intended only for parents of children aged 6 years or younger. The six-year mark was chosen because, at this particular age, children begin significantly developing their ability to recognise and correctly interpret dog signals. This would indicate that children six years of age and younger are at the greatest risk of finding themselves in a potentially dangerous situation with a dog. If the questionnaire was filled in by a respondent who did not meet this criterion, their responses were automatically discarded. Introductory questions regarding the age and sex of each parent were followed by questions regarding the presence of a household dog/dogs (dog owner status) and the number of children in the household. The questionnaire was freely accessible; the respondents could also send each other a link to fill it out.

The survey included 15 photographs depicting 5 different interactions, all of which were considered potentially dangerous, with each interaction depicted once with each of the above-mentioned 3 dogs. The respondents were asked to select which photos they perceived as indicating a potentially dangerous interaction between the dog and the child. We chose to depict the following five specific situations:

1. Next to a toy: the child is sitting on a couch next to the dog, which is lying on its cushion. The dog’s toy is also on the cushion, and the child is not making physical contact with the toy.
2. Touching a toy: the child is sitting next to the dog, which is lying on its cushion, and the child is touching the dog’s favourite toy, which is between the child and the dog.
3. Hugging: the child and the dog are both sitting on a couch, with the child hugging the dog tightly around the neck.
4. Staring into the dog’s eyes: the child is kneeling in front of the dog and staring into its eyes without touching the dog.
5. Touching a bowl: the child is touching a bowl of dog food, with the dog in close proximity.

**Figure 1:**
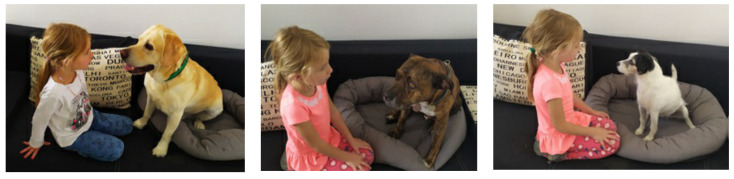
Tested situation called ‘Staring into the dog’s eyes’ with the Labrador (**left**), Pit Bull (**center**), and Russell (**right**).

Respondents were allowed to select one, two, or all three of the photos for each question (Supplementary Figure S1). The fourth tested situation ‘Staring into the dog’s eyes’ with all three dogs is captured in Figure 1.

### 2.5. Testing the Questionnaire

The questionnaire was first tested on a group of 12 respondents. These initial questions were somewhat unclear, and they were subsequently reworked and successfully retested on another group of 12 respondents, who did not take part in the final survey. 

### 2.6. Statistical Analysis

Calculations were performed using the GLIMMIX procedure that fit a logistic linear model (Statistical Analysis System, Version 9.3, 2018). Here, the dependent variable (*yi*) ‘risk assessment’ has a value of 1 (risky situation) with a probability of π*_i_* or 0 (not-risky situation) with a probability of 1 − π*_i_* for observation *i*. We used the following model to determine the probability of risk assessment:

$$log\left(\frac{\pi_{ijkl}}{1-\pi_{ijkl}}\right)=Sex_{i}+Age_{j}+Dog\;owner_{k}+Nb\;of\;children_{l}+Dog\;Type_{m}$$

where *Sex_i_* is a fixed effect of the *i*th Sex (*i* = men or women); *Age_j_* is a fixed effect of the *j*th Age (*j* = 21–30; 31–40; 41–50); *Dog owner_k_* is a fixed effect of the *k*th Dog owner (*k* = yes or no); *Nb of children_l_* is a fixed effect of the *l*th Nb of children (*l* = one, two, three, four); *Dog Type_m_* is a fixed effect of the *m*th Dog type (*m* = ‘Labrador’, ‘Pit Bull’, ‘Russell’). 

The effect of the type of situation was tested by the following model:

$$log\left(\frac{\pi_{ijkl}}{1-\pi_{ijkl}}\right)=Sex_{i}+Age_{j}+Dog\;owner_{k}+Nb\;of\;children_{l}+Dog\;Type_{m}\;\;+Type\;of\;Situation_{n}$$

where *Sex_i_*, *Age_j_*, *Dog owner_k_*, *Nb of children_l_*, and *Dog Type_m_* are defined in the model above, and *Type of Situation_n_* is a fixed effect of *n*th class of situations (*n* = ‘next to a toy’, ‘touching a toy’, ‘hugging’, ‘staring into dog eyes’, and ‘touching a bowl’).

Least-squares means of analysed effects (LSMEANS) in the presented model were estimated on the logit scale and subsequently back-transformed to the original scale (probability) using the following inverse link function:

$$\pi_{ijkl}=\frac{\exp\left(LSMEANS\right)}{1+\exp\left(LSMEANS\right)}$$

Differences between the least-squares means were tested at the significance level (error probability) of *p* < 0.05.

## 3. Results

### 3.1. Differences between Situations and Dog Type

**Figure 2:**
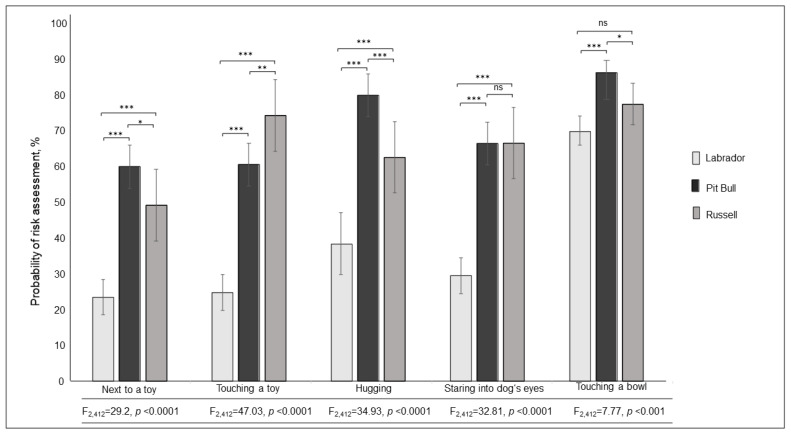
Differences between probability of risky situation assessment in the photo-set of all dogs. LS means ± S.E. * *p* < 0.05, ** *p* < 0.01, *** *p* < 0.0001, ns = *p* > 0.1.

The probability of risk assessment was significantly affected by the presented situations (F_5,3715_ = 30.2, *p* < 0.0001) and dog type (F_2,3715_ = 96.72, *p* < 0.001); see Table 1. Later, we analysed every situation separately and found that dog type had a significant effect on risk assessment; the respondents consistently selected the Labrador as the least dangerous dog; however, respondents rated the other two breeds differently for each situation with the exception of one, in which they provided a similar evaluation for both dogs (Figure 2).

### 3.2. The Effect of Age and Dog Ownership

**Figure 3:**
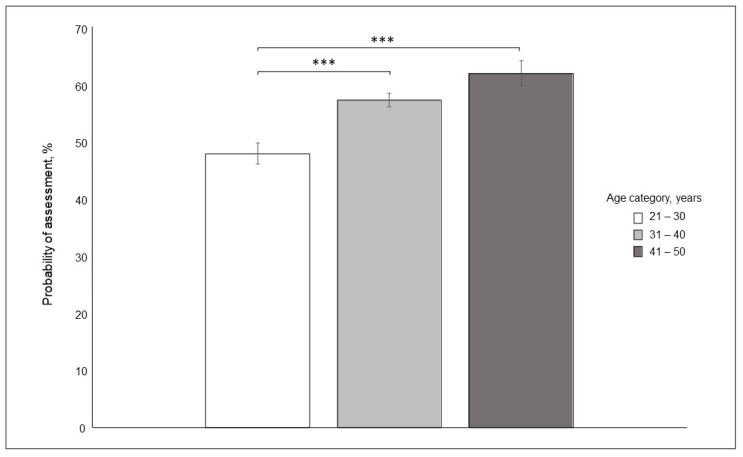
Differences between probability of risky situation assessment between age category of the respondents. LS means ± S.E., *** *p* < 0.0001.

We found that the youngest respondents (21–30 years old) were significantly less likely to assess a potentially dangerous situation than were respondents from both the 31–40 and 41–50 age categories (Figure 3; F_2,3715_ = 13.69, *p* < 0.001).

Respondents who were also dog owners were significantly more adept at successfully assessing a potentially dangerous interaction than were respondents who did not own dogs: 59.11% ± 1.2 S.E. for dog owners vs. 52.8% ± 1.8 S.E. for respondents without dogs (F_1,3715_ = 9.75, *p* < 0.01). According to the statistical models, neither the sex of the respondent nor the number of children per household had any significant effect on the probability of assessment (data not shown). The frequency distribution of categorical fixed effects is presented in the Supplementary Files (Supplementary Table S1).

## 4. Discussion

In this study, parents tended to downplay the possible risks to the child in four of the five presented situations (between 58% and 68% of respondents assessing these situations as potentially dangerous). Only the last situation (touching a bowl) was deemed potentially dangerous by nearly 80% of respondents. This was in accord with our hypothesis that participants of this study would fail to see every presented situation as potentially dangerous. More importantly, this study indicates that the type of the dog seems to be a more important factor than the situation itself when assessing the possible dangers of child–dog interactions. As expected, respondents felt the Labrador posed less of a danger than that of the Pit Bull (in all situations). Moreover, respondents considered the Russell (the smallest dog) more dangerous than the Labrador in all situations, which was contrary to our hypothesis.

### 4.1. Assessment of Situations and Types of Dogs

Regardless of the type of dog in the presented situations, the respondents considered ‘next to a toy’ and ‘touching a toy’ situations less dangerous (less than 50% perceiving a risk) than ‘hugging’ and ‘staring into the dog’s eyes’ situations (less than 60% perceiving a risk). Parents tend to allow children to interact freely with dogs as long as they see those interactions as being ‘amicable’ [33], meaning that the parent or guardian might overlook any potential danger. This was clearly indicated with regard to the first two situations (‘next to a toy’ and ‘touching a toy’), in which the Labrador was consistently seen as posing little threat, while the Pit Bull and Russell were considered potentially dangerous by at least half of the participants in both situations despite the fact that child–dog interactions involving a toy are common backdrops of dog attacks [11,32].

The situation termed as ‘touching a bowl’ was perceived as the most dangerous regardless of breed (almost 80% of respondents shared this view). One explanation for why this situation was rated as potentially dangerous regardless of dog type might be that common sense generally dictates that one should not initiate contact with or approach a feeding dog. Some respondents might have, at some time in their past, heard of or witnessed a dog attack that was initiated by a person touching a dog’s bowl [49].

Situations termed as ‘hugging’ and ‘staring into the dog’s eyes’ involving the Pit Bull and Russell were assessed as dangerous by a majority of participants. However, any potential danger posed by the Labrador was, again, thoroughly underrated (with a maximum of 40% of respondents seeing the Labrador as a potential threat). Although it is known that dogs may use eye contact as a means of intimidation [56,57], people may not immediately perceive any danger in this behaviour [58] since it is not normally an indication of aggression among humans [59]. This result reveals that some parents or guardians might unknowingly expose their children to the risk of harm from a dog simply by not realising that something as natural as establishing eye contact might, under some circumstances, be perceived as a threat even by a dog that is deemed harmless. Any potential danger is elevated if the situation involves a dog of a highly regarded family breed such as the Labrador Retriever. Both of the above-mentioned situations are known to be a common backdrop of child-directed dog attacks [31]. Guardians or parents should always be significantly concerned in situations when a child’s face is in close proximity to that of a dog.

### 4.2. Effect of Respondent Age

Younger participants (21–30 years) were less likely to assess a potentially dangerous situation from the photographs, by a margin of 10%, than were respondents from both the 31–40 and 41–50 age categories. The results indicate that older parents of young children tend to be more attuned to potential dangers than their younger counterparts. These findings may be in agreement with those of Clutton-Brock [60], which indicate that older mothers who are near the end of their reproductive years provide their offspring with greater levels of maternal care than younger mothers, even at the cost of jeopardising their chances of future reproductive success or survival. Similarly, Tearne [61] suggested that children of older mothers are subject to greater protective care, which affects their behavioural and cognitive outcomes.

### 4.3. Dog Ownership Effect

Participants that owned dogs were more likely to evaluate all situations as more dangerous than were participants that did not have a dog at the time of this study; however, the difference between these two groups was minimal (59% respondents with dog ownership and 53% respondents who did not own dog). Therefore, we are inclined to believe that such a tentative effect has little to no practical use in correctly interpreting dog behaviour under these circumstances. This view is in line with previously published findings on the topic of dog–human communication signals [18,19,22,23,62]. One possible explanation for the dog-owning respondents not marking certain interactions as potentially dangerous is that they routinely tend to underestimate any danger posed by their own dogs [3]. However, the respondents were questioned only about their current dog ownership status (at the time of the survey) rather than any past experience they might have had with dogs.

### 4.4. Limitations

The survey was distributed with no limits to respondents except that they had to be parents of a child aged 6 years or under. Equal sex or gender distribution was not the focus and could not be attained; hence, the overwhelming majority of respondents were mothers of young children. A significant effect of the sex of respondents was not revealed; however, because of the discrepancy in numbers of men and women, we cannot rule it out.

The survey did not investigate the respondents’ level of experience with dogs or their approach to dogs. A more focused exploration of their personal experience with dogs might reveal an effect it could have on their perception of child–dog interactions.

The choice of dog breeds included in the survey was influenced by key factors: The individuals had to collaborate during the photography sessions, and the breed had to be commonplace in the entire Czech Republic, while also being mentioned in scientific literature. However, a wider breed selection and a more robust dataset would be beneficial to identify what factors are important in child–dog interaction risk assessment.

We consider this to be a pilot study that could be a baseline for further, detailed human–dog interaction research.

## 5. Conclusions

Our respondents consistently viewed the Labrador as posing significantly less risk than the Pit Bull and even the Russell, which was initially hypothesised to be seen as less dangerous. The only instance in which the Labrador was seen as potentially dangerous was the situation termed ‘touching a bowl’. Parents aged 31 years and older perceived all situations as riskier than did respondents between 21 and 30 years of age. Participants who owned dogs described the situations in the photos as slightly more dangerous than respondents who did not own dogs; however, this effect did not seem to be of much importance. Although records indicate that Labrador Retrievers are not generally involved in a high number of serious human-biting incidents, underestimating the potential dangers of a dog-child interaction regardless of the breed is ill advised. Additionally, based on our findings, we recommend including the parents, guardians, or teachers of children aged 6 years or younger in educational programs and activities focusing on child–dog interactions. Such programs should emphasise that when assessing risk in situations involving children and dogs, the nature of the interaction should be taken into consideration more so than the breed of dog.

## Figures and Tables

**Table 1:** Differences in the probability of the risk assessment between dog types (upper section) and various situations (lower section). Data are presented as LS means ± standard error included *p*-values.

Risk Assessment					
Dog Type ^1^	Labrador	Pit Bull	Russell		
Probability, %	39.1± 1.6 ^a^	65.0% ± 1.6 ^b^	63.3% ± 1.6 ^c^		
**Situations**	**Next to a toy**	**Touching a toy**	**Hugging**	**Staring into the dog’s eyes**	**Touching a bowl**
Probability, %	48.4 ± 2.2	48.7 ± 2.2	56.0 ± 2.1	56.5 ± 2.1	77.9 ± 1.8
	***p*** **-value ^2^**				
Next to a toy	-	ns	0.01	0.01	0.0001
Touching a toy		-	0.05	0.01	0.0001
Hugging			-	ns	0.0001
Staring into dog eye’s				-	0.0001

^1^ Differences between categories of the fixed effect ‘dog type’: ^ac^ *p* < 0.0001, ^ab^ *p* < 0.0001, ^bc^ *p* < 0.05. ^2^ Differences between categories of the fixed effect ‘situations’.

## Data Availability

The data presented in this study are available on request from the corresponding author. The data are not publicly available due to protection of privacy of respondents, as the dataset is part of a larger survey that contains additional respondent information.

## Supplementary Materials

The following supporting information can be downloaded at: https://www.mdpi.com/article/10.3390/ijerph19010564/s1, Figure S1: A list of photographs offered to respondents in the questionnaire by individual situations (in order from left the Labrador, the Russell, and the Pit Bull), Table S1: Frequency distribution of categorical fixed effects from 207 participants.
