# Supplementary material for: How Parents Perceive the Potential Risk of a Child-Dog Interaction
Source: Int J Environ Res Public Health. 2022 Jan 5;19(1):564. doi: 10.3390/ijerph19010564 (PMC8744742; doi:10.3390/ijerph19010564)

Figure S1: A list of photographs offered to respondents in the questionnaire by individual situations (in order from left the "Labrador", the "Russell" and the "Pit Bull").

1. *Next to a toy:* the child is sitting on a couch next to the dog, which is lying on its cushion. The dog's toy is also on the cushion, and the child is not making physical contact with the toy.

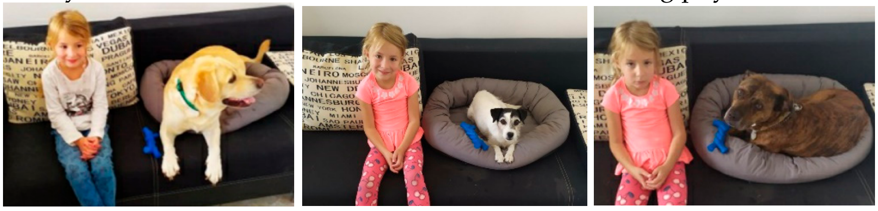

2. *Touching a toy:* the child is sitting next to the dog, which is lying on its cushion, and the child is touching the dog's favorited toy, which is between the child and the dog.

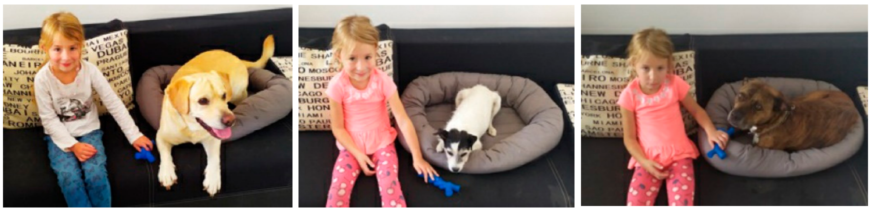

3. *Hugging:* the child and the dog are both sitting on a couch, with the child hugging the dog tightly around the neck.

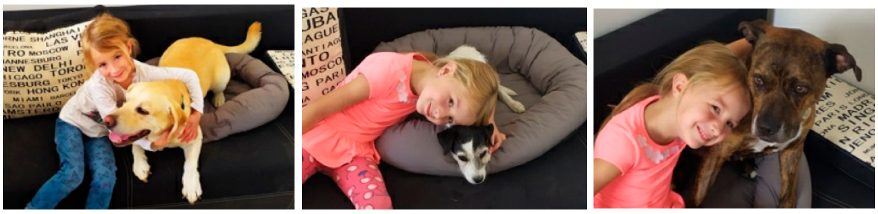

4. *Staring into the dog's eyes:* the child is kneeling in front of the dog and staring into its eyes without touching the dog.

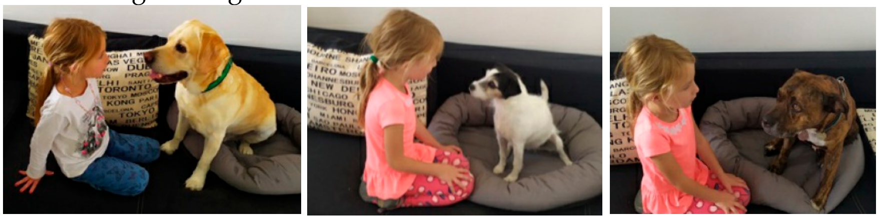

5. *Touching a bowl:* the child is touching a bowl of dog food, with the dog in close proximity.

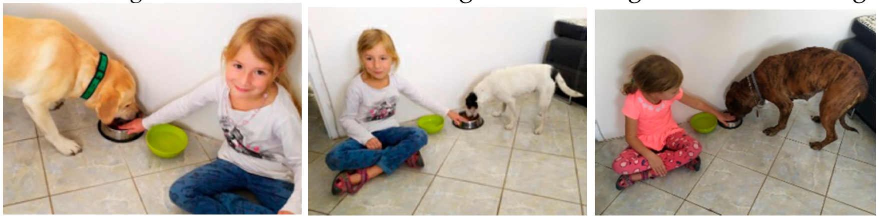

Supplement: Supplementary file 1 [file ijerph-19-00564-s001.zip › Supplementary Figure S1.pdf]
