# Supplementary material for: How Parents Perceive the Potential Risk of a Child-Dog Interaction
Source: Int J Environ Res Public Health. 2022 Jan 5;19(1):564. doi: 10.3390/ijerph19010564 (PMC8744742; doi:10.3390/ijerph19010564)
Supplement: Supplementary file 1 [file ijerph-19-00564-s001.zip › Supplementary Table S1.pdf]

Fixed effects	Frequency	Percent, %
**Gender of participants**		
Male	14	6.76
Female	193	93.24
**Age of participants**		
21-30	44	21.26
31-40	133	64.25
41-50	30	14.49
**Ownership of dog**		
Yes	158	76.33
No	49	23.67
**Number of children**		
1	88	42.51
2	85	41.06
3	31	14.98
4	3	1.45

Supplementary Table S1: frequency distribution of categorical fixed effects from 207 participants
